# Interdisciplinary discussions on palliative care among university students in Spain: giving voice to the social debate

**DOI:** 10.1080/17482631.2021.1955441

**Published:** 2021-08-06

**Authors:** Carla Reigada, Santiago Hermida-Romero, Anna Sandgren, Beatriz Gómez, Inés Olza, Alejandro Navas, Carlos Centeno

**Affiliations:** a ATLANTES Research Group, Institute for Culture and Society, University of Navarra, Pamplona, Spain; b Navarra Institute for Health Research (Idisna), Pamplona, Spain; c PhD Student in the Applied Creativity School, University of Navarra, Pamplona, Spain; d Center for Collaborative Palliative Care, Department of Health and Caring Sciences, Linnaeus University, Växjö, Sweden; e School of Communication, University of Navarra, Pamplona, Spain; f Emotional Culture and Identity Project, Institute for Culture and Society, University of Navarra, Pamplona, Spain

**Keywords:** Palliative care, students, perceptions, education, social debate

## Abstract

**Background:**

University students are the future professionals who will influence society. It is thus essential to improve students’ understanding of palliative care (PC), especially those in the non-health field, to generate and encourage propitious opinions about PC. This study describes the perceptions of PC among university students from different disciplines.

**Method:**

Qualitative exploratory study using virtual focus groups (FGs) and design thinking (DT) approach. An intentional sample of students from various disciplines, universities and cities from Spain were selected. Numerous researchers from different fields were involved in the discussion of the final thematic analysis.

**Results:**

Four themes and seven subthemes were found: i) Students have an ambiguous understanding about PC; ii) PC is not a common issue for non-health students; ii) Students see PC as an important and necessary field; iii) Students build counter-narratives against PC myths, demonstrating PC brings key benefits into people’s lives; iv) PC is a synonym of death.

**Conclusion:**

Despite the fact that students do not know much about PC, the topic easily arouses reflection and positive discussion. Through the conversations they find positive values and arguments against misunderstand- ing. Students from different disciplines could be the target of innovative educational initia- tives and the social debate on PC.

## Background

Palliative care (PC) offers a holistic approach to improve the quality of life among people facing advanced and incurable diseases (Radbruch et al., 2020). This concept was defended by the World Health Organization (WHO) in the 1990s, yet today several studies show that the misunderstanding of this medicine persists among the general public, health professionals, and policymakers (Centeno et al., 2017; WHO, 2014). One problem is the method used to transfer knowledge, which often inadequately reflects the real practice of PC (Carrasco et al., 2017). For this reason, end-of-life and PC education and training has become a priority in many European countries (Martins Pereira et al., 2020; Paal et al., 2019).

Many studies describe how undergraduate educational programms can improve positive attitudes and knowledge about PC. As a result, many universities have developed basic PC programms to educate future professionals, although almost exclusively for students of medicine and nursing (Noguera et al., 2018). Studies on empathic understanding by university students on PC concluded that students from health disciplines have high levels of sensitivity towards this topic (Ballesteros et al., 2014; Centeno et al., 2016) but lack specific knowledge and skills, continuing to manifest negative attitudes (Barclay et al., 2015; Dimoula et al., 2019; Jiang et al., 2019). PC continues to be excessively associated with the image of “death”, “terminal illness”, or “end of life”, and is rarely associated with values such as “compassion”, “dignity”, or “quality of life” (Shalev et al., 2018).

Students from different disciplines had little knowledge of the subject: many thoughts that PC is about end-of-life and, consequently, those with less knowledge and interest in end-of-life issues were more likely to have negative attitudes towards PC (Miltiades, 2020). Older students and female students reveal a more positive attitude, which they believe is promoted through experience or vital contact with this theme instead of studying about it (Kirkpatrick et al., 2019).

Studies on students’ perceptions of PC are almost entirely focused on students in health care fields; however, students from other fields (e.g., social work, economics, business, communication, education) are also part of the future world of professionals. However, it is important to study both groups, not comparing these groups, but giving both the opportunity to be actively involved in PC research and self-learning.

This study aims to explore the perceptions on palliative care among university students from different courses. It is a diagnostic study that will inform a larger action-research study: 1^st^) a social intervention program with students to design a transversal PC discipline (employing a public engagement approach), and 2^nd^) the implementation of the discipline across all faculties in the University of Navarra (Spain). Ultimately, this will allow students to recognize the care of the person in PC as a matter of social responsibility.

## Method

### Study design

Qualitative exploratory study using virtual focus groups (FGs) as the main method of collecting data. The FGs aimed, collectively, to get information in an interactive way through a focused discussion. The number of participants and FGs needed was determined by the appearance of new data until a saturation point was reached (Hudson, 2003; Morgan et al., 1998). This method was combined with Design Thinking (DT) methods, facilitating the dynamics within the group, creatively to ensure a person-centred discussion based on a particular problem (Altman, Huang & Breland, 2018). DT is an empathy-focused systematic approach, a novel methodology not often applied in PC studies. The combination of these two methodologies was implemented in studies that mainly aimed to develop solutions focused on complex problems using user-centred design and with a social innovation approach (Randall et al., 2020; Smith & Phillipson, 2020). Both methodologies adopt the same cycle of actions: 1. empathy/definition or diagnosis; 2. idea or planning; 3. prototype or action; 4. testing or evaluation 5. learning and refining a new action or prototype. Both require multidisciplinary teams to generate multiple perspectives on an issue. To our knowledge, the combination of these creative methodologies could be useful to do research in PC because it promotes understanding and fast visualization of the data, making the themes progressively explicit, and reflecting on them throughout the process (Bazzano et al., 2017). We assume a constructivist perspective in that the existence of multiple realities can result from human construction and where, interactively, it is possible to obtain factual results. Through a process of constant analysis, the intersubjective interpretations can be discussed and achieve a reality valid to be explained in a given context (Denzin & Lincoln, 2013).

This project adopted the following concepts: PC is “an active holistic care of individuals across all ages with serious health-related suffering due to severe illnesses, and especially of those near the end of life” (Radbruch et al., 2020); the perception is understood as the behaviour created in interaction with others based on preconceptions, feelings, knowledge, thoughts, and expectations of each person (Skinner, 2009). This study corresponds to the diagnostic phase of a broader research project to implement a pilot palliative social intervention programms in the University of Navarra in Spain.

### Participants

Between June and August 2020, university students from throughout Spain were invited to register and participate in a virtual FG to discuss PC. The call was open to any student from the first, second, or third year of clinical and non-clinical degrees, up to 22 years of age, who were able to understand and speak Spanish, not with intention to compare these groups but giving both the opportunity to be actively involved in PC research and self-learning. It was promoted on social media at the national level, and through the collaborators. The study was promoted through an original poster created by students of the design school and highlighted that the meetings would be zoomed in, using creative techniques of DT and that the participants would have a voucher of 10€ amazon as a reward.

Twenty students participated in three focus groups (FG1 = 7; FG2 = 6; FG3 = 7), six of whom took part in a fourth FG. The mean age was 19 years old (range 18–22) and 14 students were women. The students were from seven different communities of Spain (Navarra = 12, Galicia = 1, Madrid = 1, Aragón = 3, Basque Country = 1, and Valencia = 2), 14 studied at private universities, and students were enrolled in both health degree (n = 9) and non-health degree (n = 11) programs. All students have had contact with the subject: some had ill close relatives (n = 8), and others heard about it in the news or through friends (n = 12).

### Data collection and analysis

Whenever a student registered for the study, they received an email with specific information, an informed consent, and the details of a virtual FG. Three FGs were run on different days. Each FG lasted approximately two hours and was facilitated by two project researchers: one expert in DT (SH) and the other in social sciences and PC (CR). The FG was video-recorded, transcribed, and analysed (see Appendix I—FG interview guide).

The FG transcriptions were used to conduct a thematic analysis, inductively, with no predefined codes, by two researchers from different fields (social work and nursing) with experience in qualitative analysis and PC (CR and AS), paying attention to differences. The preliminary categories and themes were discussed with the other three researchers (SH, BG, and CC), experts in DT, narrative journalism, and palliative medicine, paying attention to differences (Braun & Clarke, 2006). At the same time, an empathy map was created in Miro software to dynamize the groups [21]. The empathy map is a visual tool that aims to establish a quick and visual record of the most discussed topics in each group. It represents “what students feel and think” (preconceptions), “what students hear” (preconceptions, feelings), “what students see” (knowledge), and “what young university students say and do” (thoughts and expectations) concerning PC. This information was used for the final discussion of the thematic analysis among the four researchers (CR, AS, SH, BG).

The preliminary findings were presented by CR and SH during a fourth FG, which integrated two students from the three original FGs to obtain their feedback and enhance trust. Notes were taken from this session, and it was analysed and discussed with the other two researchers (AS and BG).

Also, the dynamics of the three FGs were analysed by two independent researchers, experts in linguistics (IO) and observational studies (CR), using the Interactional Discourse Lab software (https://interactionaldiscourselab.shinyapps.io/IDLab/) to represent and understand the dynamics and visualize which topics were most discussed among the students.

All data was analysed with the following research question in mind: “What do students perceive and feel about palliative care?” This guided the presentation of the results, showing the rationale and the emotional aspects of the perceptions about PC (thoughts, knowledge, feelings, preconceptions, expectations) of university students. The trustworthiness of the research findings was achieved by triangulation (multiple methods, multiple researchers view-points and multiple type of data) allowing to explore different sides of the problem and contributing to a consistent interpretation of the data.(Table I)

**Table I.** An example of the thematic coding

Research question: What do students perceive and feel about palliative care?			
Data	Code	Category	Themes
“*I identify it more with personal care … it is a more detailed attention … I mean when extra support is needed … ”*	personal care detailed attention extra support	Conception Definition	Understanding Knowledge

### Ethical approval

This study was approved (No. 2020.120) by the Ethics Committee of the University of Navarra. All participants provided their written consent for the study.

## Results

(You can find the resume of the results in Supplementary Material)

### Speech frame: palliative care is synonymous with death

Students transmitted their ideas on the topic of PC using two terms interchangeably: “palliative” and “death”. This indicated that, in students’ minds, these two words were the same. For this reason, we assume that this study entails a speech frame that is transversal to all themes presented below. It would be beneficial if, in a future study, these two concepts could be discussed separately with university students.

### Ambiguous understanding about PC

#### What students know

Various levels of knowledge on PC were identified, describing what PC is, to whom and when it is addressed, and what it provides. Students identified PC as follows: support, “*Treatment intended to relieve pain when a patient is in a terminal phase*” (P5S1).; personal care, “*I identify it more with personal care … it is a more detailed attention … I mean when extra support is needed … ” (P4S1)*.; and a psychosocial measure, *“I believe that PC includes all the health and psychological measures that help a person to go through a situation when it is already complex … ”(P3S1)*. Students perceive PC is for terminal and non-terminal patients who are very ill, for patients with cancer and their families, and for those who have no cure. In general, they think people receive PC at the end of their lives: *“It may be that it is not for a hundred percent of terminal patients, but that treatment is started earlier, not only at the final stage”, “ … and for example, I thought it was only for terminal patients and it is not. If you are diagnosed with cancer, they can call the palliative care team to be with you” (P5S1).*

The students believed PC provides a better and more dignified ending, decreases suffering, and supports patients in a medical and emotional/psychological way. Also, PC teams can medicate patients who are in pain, assist families by giving both hope and a positive atmosphere, and can be provided at home. The students emphasized PC gives patients back the status of a person:

I did not have a very clear idea of palliative care, but I did understand that it helps to increase the well-being of both you and your family, making the terminal illness of the patient lighter. (P2S2)

How to stop being ‘the patient’, the poor thing, even while they are taking care of you, you can talk, have contact … and go back to being a person, and not the poor terminally ill person who is at home. (P5S3)

#### Provoking curiosity and reflexivity

It seems PC is not an unnoticed issue. Although always transmitting the idea that they did not have too much knowledge about PC, students asked many questions concerning specific roles of PC during the FG discussions, which reflects interest in the subject when it is discussed within a group of people. Discussions about it invited both immediate and more long-term reflections. Students confirmed that they had thought more about the topic after the first FG. Questions that were raised by the students about PC includes the following:

Are they (PC) free? Can your GP refer you to this type of care?’; ‘Speaking fast and badly, what can PC offer me (specifically)? Conversation, answers, and medicine?’; ‘Are there people who leave PC programs before they die?. (P3S3)

### PC is not a common issue for non-health students

#### Students do not talk about PC

Students do not talk about PC issues in their daily lives. On the one hand, it is a difficult topic that brings them anguish, and, on the other hand, it is considered a complicated and taboo subject that may require a certain maturity. For that reason, most of the time, students preferred to ignore it. They also argued this is a subject that does not fit with their youth and, frequently, they cannot talk about it in their friendship circle, at the school or university. Health-related careers students are expected to speak more frequently about this topic. However, students in non-health careers affirmed that it is the experience or previous contact with palliative situations (direct or indirect) that allows them to talk about the subject. Students discussed PC issues because of their practical training and because of their life experience:

There are many people who try to avoid it because it makes them happier … it is a complicated subject … it is a very, very difficult subject and I don’t know, a bit taboo, too’. (P1S2)

Young people do not speak of it because of fear, because in the end they are afraid of illness and death. They preferred to live in a certain ignorance on this subject and live more the daily life. (P2S1)

#### Early awareness of PC-related issues would be beneficial

Students perceived that this topic will come up at some point in their lives. They felt that students should talk about it at high schools and universities by listening to real experiences from patients, professionals, and relatives. However, talking about this topic with young people requires an appropriate educational strategy, finding the right moment, balancing the motivation, attention, and the necessity felt by young people:

In the end, we who are studying careers in the field of health, it is a topic that will surely touch us, either in practice or during our studies. It is something that we are going to have to see, but other people from other courses do not. (P1S1)

It would be a good idea for schools to talk about it, not explained by your math’s teacher, but rather a doctor who has had experience … even by a patient … this can affect you more and you will understand it better. (P3S2)

### Palliative care: An important and necessary field

#### Palliative care feels like a good thing

When known and understood, PC is recognized as a relevant and needed field for society. However, since it is understood as synonymous with death, they assume it is something good to access during unescapable moments such as when you are dying due to a serious illness. Mostly students would like to receive it or have it for their family in case of need, because the perception is that it brings a higher quality of life:

And why does medicine have to serve only to extend life without trying to make it more comfortable? I would not like to think that my mother, for example, could leave this world with such suffering. I would like to receive it (PC) if I need it and if any family member needs it. (P6S3)

#### The real information about PC does not reach students

Everyone sees PC as a good thing *per se*; however, this does not justify why it is not a topic with much interest in the society at large. PC does not provoke a political debate as in discussions about euthanasia. Students perceived that if there is no public debate on an issue, you do not think or talk about it. It is as if PC does not exist or, you do not realize the lack of it. Even though these events took place in the midst of a global pandemic where suffering and death are a reality, PC is not a topic discussed broadly in the media, though it should be:

I think, for example, that even in this coronavirus situation, with millions of patients in palliative treatment and being terminal, no concrete news has really come out about their process. It could have been a good opportunity to learn about this … But even so, no one has heard of it. (P7S3)

I also believe that euthanasia provokes greater debate, being in favor of euthanasia or against euthanasia, similar to abortion. On the contrary, PC does not create much debate: there are no people who are for or against. (P3S1)

### Positive message: PC brings key benefits into people’s lives

During the FGs, students did not limit themselves to commenting on PC. As they discussed the issue, they assumed it as something they had to defend (like a proclamation). They explicitly built counter-narratives offering a positive message of PC, deconstructing the myths and misunderstandings of the current negative discourses that characterize PC.

University students perceive that PC can bring key benefits into people’s lives, including the following: i) meaning, in the sense that psychological attention is applied and suffering is reduced; ii) normalization, making patients feel like people again and helping them to have as normal a life as possible; iii) facilitation, because caregivers allow and facilitate difficult conversations; and iv) peace, as PC helps people die in peace:

I would expect a lot of psychological attention from the PC … I really like what you said about difficult conversations. It’s something that I would really appreciate. (P7S1)

They (PC) try to make the situation as normal as possible, since you know that you are going to have to live with the disease. They help you to make your life change as little as possible and to move forward with it in the best way possible. (P5S2)

### The topic discussed and the dynamics in the FG

**Figure 1.**
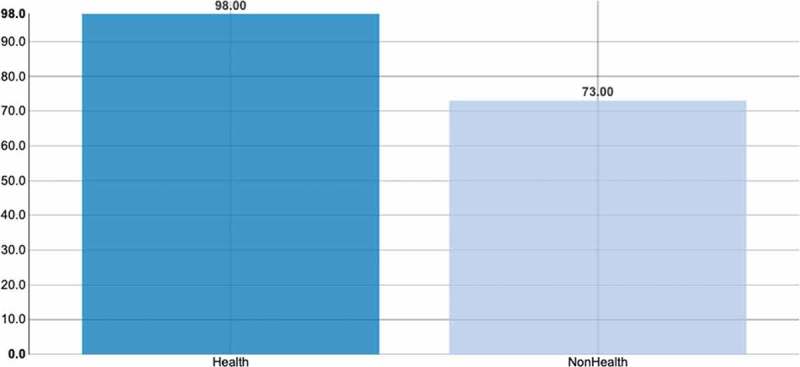
Level of participation in the FG

**Figure 2.**
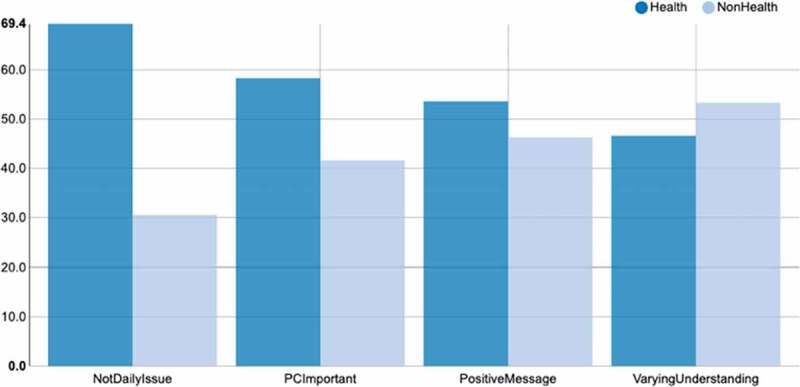
Speaker proportion for each tag

The students from health disciplines were more active in the discussions and expressed greater concern about the fact that PC is not a common or daily theme. Both groups spoke in the same way about the importance of PC and both contributed with positive counter-narratives about PC (Figures (1, 2)).

Bar chart of each group of speaker’s participations, in terms of turns.

Proportional speaker participation for each tag. The bars for each tag sum up to 100%.

**Figure 3.**
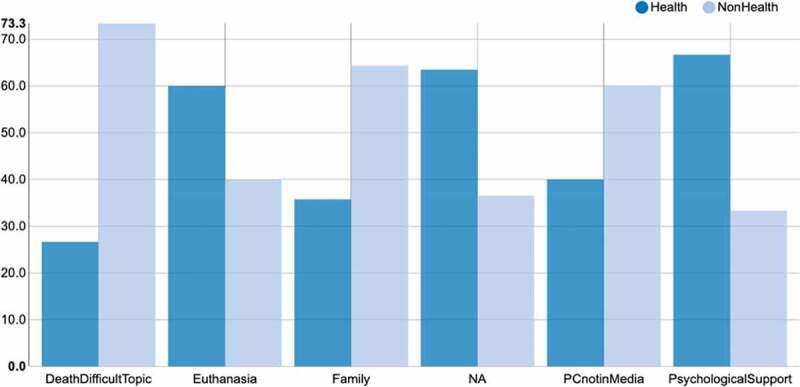
Frequency of themes mentioned by health and non-health participants

Taking as an example the dynamics of FG 2, since it is the group with a similar number of students from health (n = 4) and non-health disciplines (n = 5), we can verify that students from the health field introduced more new topics into the discussion, thus being more in command of the conversation dynamics. Also, these students were able to talk about different topics such as family, psychological support, euthanasia, among others, while students from non-health disciplines posed fewer new topics. However, students from non-health programs argued for longer periods of time. Students from non-health disciplines also tended to talk more about death and dying, and they highlighted issues related to the lack of awareness of PC in the media (Figure 3).

*Proportional speaker participation for each tag. The bars for each tag sum up to 100%*.*NA = other topics

## Discussion

The Spanish university students interviewed tended not to talk about PC because they assumed that it is like talking about something closely related to death. This is an expected result and widely described in the literature: society associates PC with death and dying, which in turn is a taboo subject (Carrasco et al., 2019; Green et al., 2018; Westerlund et al., 2018). Students recognize that it is not a topic to discuss in their friendship circles because it brings them anguish and because the topic feels removed from their youth, unless it has touched their life experience or their academic training.

There are few studies on the perception of university students about death and dying, and the majority of it is focused on children. Some studies conducted with nursing students concluded that, during their practice, feelings of sadness and frustration prevailed following the death of a patient. This experience is often so painful that nursing students prefer not to be in contact with PC patients. For this reason, one of the recommendations is to teach students to normalize death in life (Sandoval et al., 2020; Valen et al., 2019). We believe that this idea must link to the central strategic question of how to disconnect the direct image of “death” with “palliative care”. The simple fact of creating an open environment that promotes discussion of the subject among people, using art, and fostering strategies such as active listening and education for intercultural sensitivity, can help to develop awareness about death based on values like trust, instead of fear (Nicol & Pocock, 2020; Rojí et al., 2017). A systematic review of PC education has recommended teaching as authentically as possible the patient’s experience of receiving that care. This could be the best way to communicate the positive, real, and intangible meaning of PC (Centeno & Rodríguez-Núñez, 2015).

In our study, students conveyed that they do not know a great deal about PC. It was possible to uncover that health students had a more balanced understanding of the subject. Still, both groups were able to identify concepts and talk about PC-related issues, wanting to know more about it. They recognize that they are important and essential, especially at the end of life (Al-Azri et al., 2020; Barclay et al., 2015).

It was curious to see that in our study the students did not limit themselves to discussing the issue, but to develop positive counterarguments to confront the misunderstandings that exist, with an implicit intention to push back extremist messages and the propaganda that PC is equal with death. The students also recognized the importance of PC in people’s lives: PC gives meaning, normalizes the life of the person in suffering (not the “patient”), facilitates difficult conversations, and provides peace of mind. Through direct and indirect experiences, they will detect the need to speak more about this issue in society, particularly in schools and universities and in social debates in the press.

Education at the end of life is a necessary intervention for young university students (Beccaro et al., 2015; Martins Pereira et al., 2018). Currently, PC is already taught at some universities in undergraduate courses, in medicine and nursing, but much remains to be done for this measure to be recognized and implemented in other academic fields.

A positive message about the PC is urgently needed, and the way to do it may be to talk more about it and enable access to this reality. A good example that has been gaining ground, both in research and in practice, are compassionate universities: 104 international educational institutions (high schools and universities) have already signed the compassionate schools charter, and are working on compassionate action plans for students to be involved in a caring environment. Poland, the Netherlands, Spain, and Belgium are pioneers of this approach in Europe. The lack of direct and clear information that students receive from the media does not allow society to understand the true meaning of PC. To target university students, future contributing members of our society, solutions will have to be found so that these young people understand how to deal with people with vulnerability and intense suffering due to serious and advanced diseases. This study corresponds to the diagnostic phase of the main action research study to create a palliative social intervention programms with university students.

### Limitations

Started this project in the summer months was a challenge. The three summer months of 2020 marked the beginning of the COVID-19 pandemic in Spain and we saw that information on social networks was focused in this topic. However, the students who participated were really motivated by the issue of suffering, noticeable by the health and social crises created by COVID-19. Also, it was possible to facilitate the FG in a dynamic way, using design thinking techniques virtually. Even we are aware that face-to-face interaction could have facilitated the fluency of the speeches and stimulated the participants in another way, the quality of the sessions shows the genuine interest of the participants in sharing their vision and contributing to a social transformation, giving visibility to the theme.

## Conclusion

This study shows young people do not discuss issues related to the end of life within their group of friends. However, through guided discussion and reflection, they come to understand it is a relevant and needed field for society, reducing the suffering of people with serious and advanced diseases. University students are an excellent target group to talk about palliative care. They can help to transmit positive messages and fighting myths about what is considered a human right. Schools and universities are privileged places to stimulate the humanity of students towards those in intense suffering from serious illnesses, and where palliative care matters for the social debate. More applied research is needed to test specific interventions that, in an alternative and sustainable way, can instil in students’ humanistic values and principles, focused on caring for people in vulnerable conditions.

## Supplementary Material

Supplemental Material

## Focus group script—Phase 1

**Aim**: To explore the perceptions* of students about palliative care and about how to follow and care people with severe suffering due to serious illnesses.

**1. To answer the research question: What do they understand about palliative care?**

Exploring students’ attitudes (adapted from the Palliative Care Attitudes Scale.

- Describe in 3 words what the terms “palliative care” conveys to you.

- Do you understand that Palliative Care is as important as curative care?

- Should society support palliative care?

**2.To answer the research question: what do they understand accompaniment and care for people in severe suffering due to serious illness?**

Exploring students’ perceptions—start with a clinical case and ask students to comment it.

- What kind of care are we talking about?

- Who provides it?

- For whom is it intended?

- Where is it done?

- Why is it done?

**Dynamics**: The work methodology will be based on Design Thinking.

**Facilitators**: 2 researchers of the study

**Observers**: 2 researchers of the study

**Empathy map**

* Perceptions = their attitudes, knowledge, previous ideas, beliefs, mental associations, etc.

Huertas L. Adaptación Al Español De La Escala De Actitudes Ante Cuidados Paliativos: Confiabilidad Y Análisis Factorial. Psicooncología; 2015, 12(2–3):367–381. doi:10.5209/Rev_Psic.2015.V12.N2-3.51015.
