# Supplementary material for: Interdisciplinary discussions on palliative care among university students in Spain: giving voice to the social debate
Source: Int J Qual Stud Health Well-being. 2021 Aug 6;16(1):1955441. doi: 10.1080/17482631.2021.1955441 (PMC8354019; doi:10.1080/17482631.2021.1955441)

Interdisciplinary discussions on palliative care among university students in Spain: giving voice to the social debate

(Carla Reigada, Santiago Hermida-Romero, Anna Sandgren, Beatriz Gómez, Inés Olza, Alejandro Navas & Carlos Centeno (2021) Interdisciplinary discussions on palliative care among university students in Spain: giving voice to the social debate, International Journal of Qualitative Studies on Health and Well-being, 16:1, 1955441 DOI: 10.1080/17482631.2021.1955441)

**Ambiguous understanding on Palliative Care**

# What students know

Provoking curiosity and reﬂexivity

Personal and emotional/psychosocial medical care.

Better/more digniﬁed ending. PC are mostly provided at home.

Image linked to good death, sadness and defeat.

It invites immediate and not immediate reﬂection.


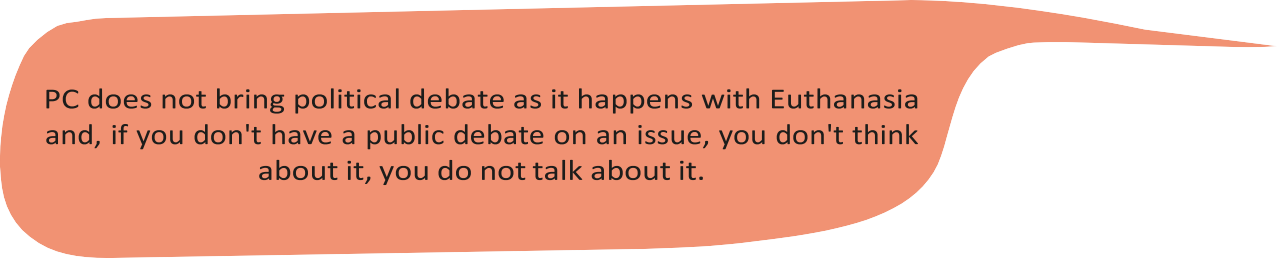

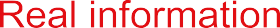


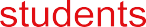


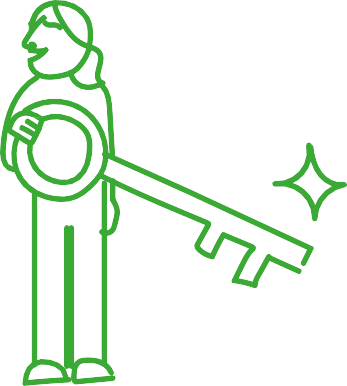

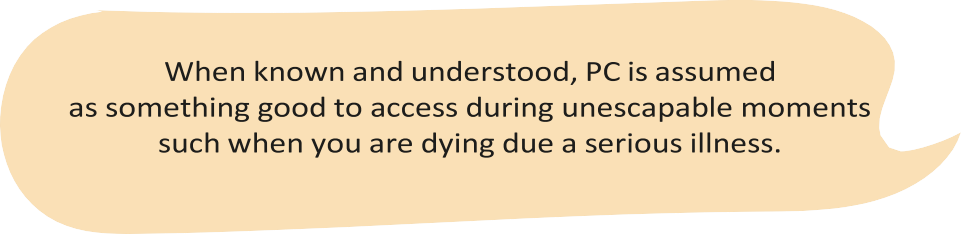
**Positive message**: **PC brings benefits into people’s lives**

PC feels like a good thing

about PC does not

**Palliative Care**: **an important and necessary field**


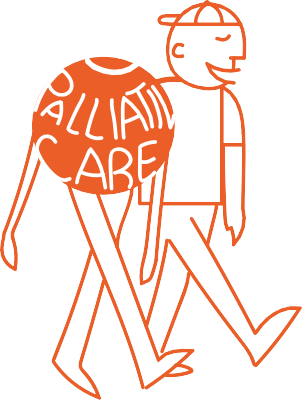


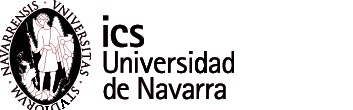

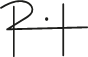

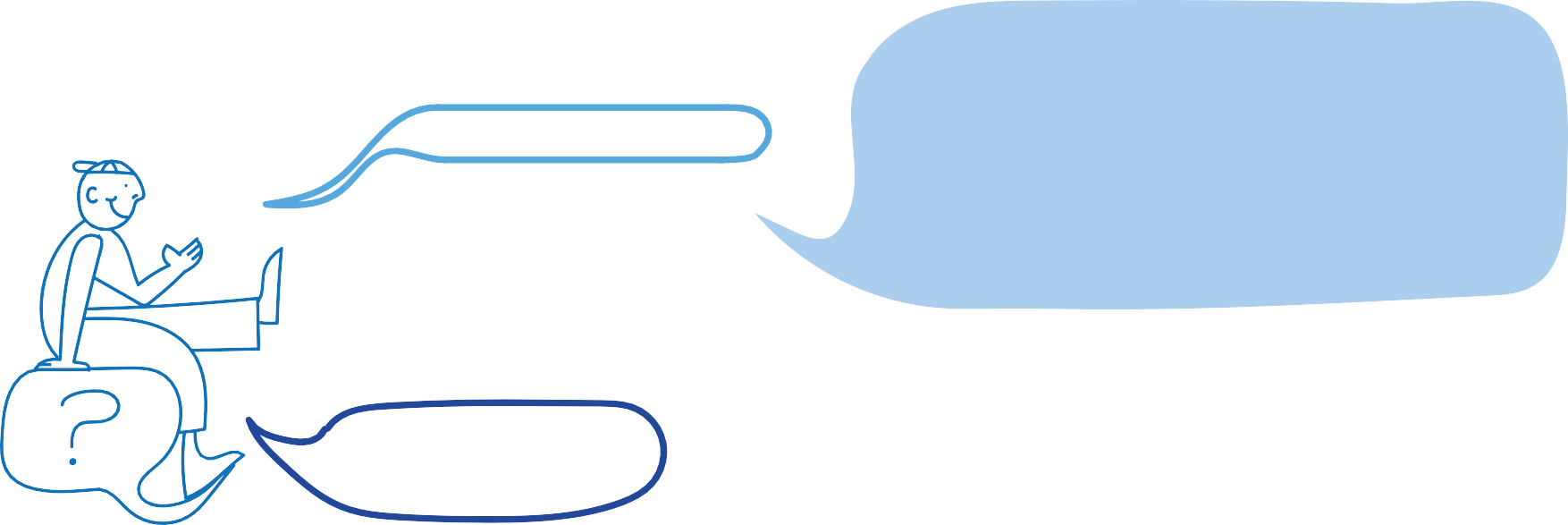

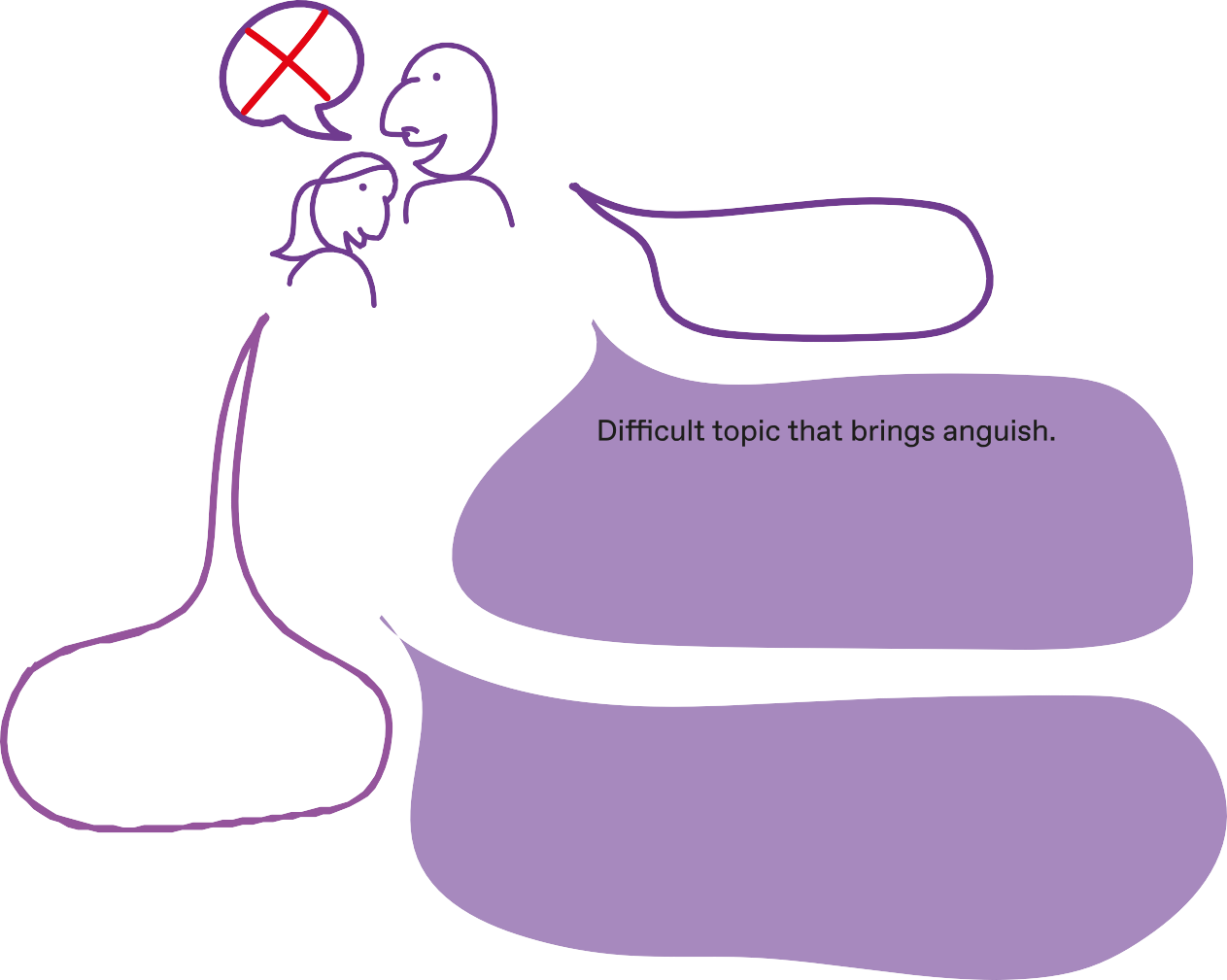

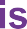

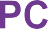

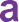

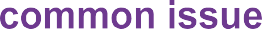

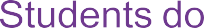

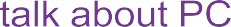

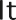

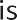

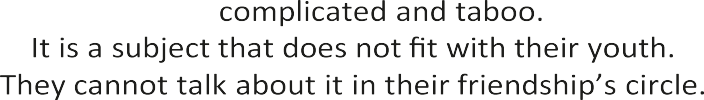

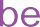

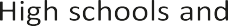

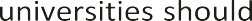

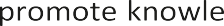

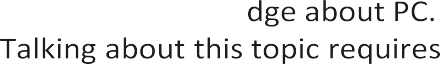

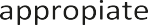

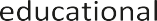

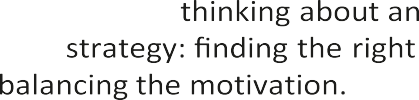

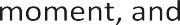


**for non-health students**

Early awareness of PC-related issues

*Speechframe:* Palliative care is synonymous with death


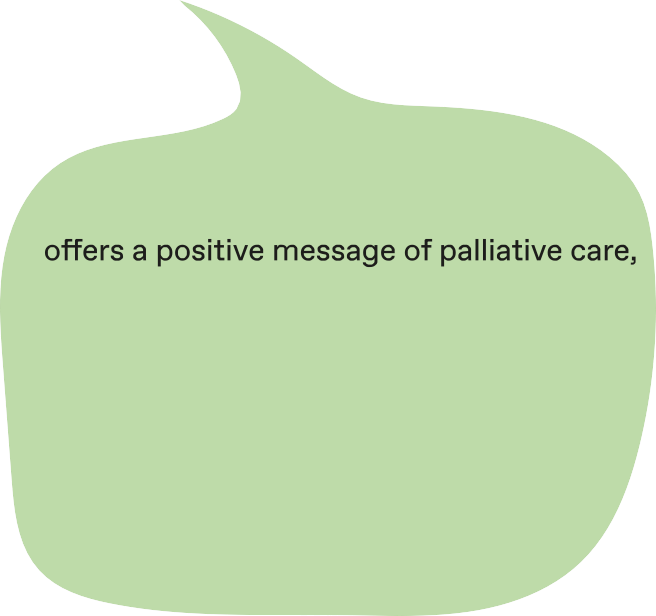

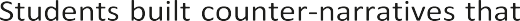

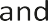

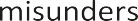

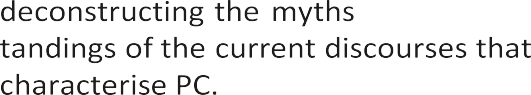

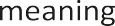

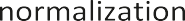

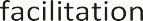

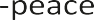

Supplement: Supplemental Material [file ZQHW_A_1955441_SM9300.docx]
